# 

**Published:** 1887-02

**Authors:** 


### ARMED FORCES MEDICAL LIBRARY

CALL NO.

Escabana

AUTHOR (*Surname first*)

TITLE

PERIODICAL

INDEPEDENT PRACTICIONER

AUG

| VOL. | NO. | PAGE | DATE |
| --- | --- | --- | --- |
| | | | 1887 |

REQUESTED BY
GEO. C. PAFFENBARGER

| SEAT NO. | DATE REQUESTED |
| --- | --- |
| 43 | AUG. 2, 1955 |

REPORT:

- NOT IN LIBRARY ☐

- NOT ON SHELF ☐

- ON LOAN ☐

- AT BINDERY ☐

- OTHER ☐

AFML FORM 19
1 NOV 54

Reserve

☆ GPO: 0—1955 324886

allowed to remain there until the wound was so far closed as to positively indicate that no suppuration existed. The capsule was then broken beneath the skin, and without exposure. In no case was there any purulent discharge. But immediately upon infection, suppuration commenced. It may therefore be considered as established, that without the presence of bacteria there can be no abscess or suppuration.

### TO SUBSCRIBERS.

THE INDEPENDENT PRACTITIONER for 1887 will be sent to the subscribers of 1886, unless we are notified that it is not desired, as it is impossible for us to open an entirely new set of books with each year. It is our pride to know that those who have taken the journal for one year usually become permanent subscribers, and are offended if it be stopped. A dental journal is a necessity for any progressive dentist, and where could such an one be better suited than with a journal that is published in his interests exclusively.

Very rarely we receive an answer to a bill sent, stating that, as the receiver had not subscribed for that year, he does not consider himself responsible for the payment. The law says that any one who receives a journal, no matter whether he shall have subscribed for it or not, is legally holden for the payment of the subscription price. If he does not wish it, his duty is clear. He should refuse it, and then the law makes it the duty of the postmaster to notify the publisher that it is not taken from the office, and failing to do this, the postmaster is himself responsible. Further, the law says that a journal need not be discontinued by the publisher until all arrearages are paid.

Of course we do not desire to send this journal to any one who does not wish to receive and pay for it, and if there be any such now on the list we hope they will notify us at once. But we as earnestly hope that there are none such.

### The Independent Practitioner—Vol. VIII.

AN INDEPENDENT DENTAL JOURNAL

### PUBLISHED BY DENTISTS FOR DENTISTS.

### Prospectus.

THIS JOURNAL is published by an association of professional men who have no other object than to offer to the profession an independent, outspoken Journal, which shall be the exponent of higher aims and a better practice.

Having no connection with any school, clique, or advertising firm, it will be impartial in its judgment of professional matters, and its appeals for support are directed to those who believe that a liberal profession should have an independent literature.

While not neglecting practical details, it will pay especial attention to those oral diseases that have usually been under the care of the general practitioner, but which more properly belong to the specialty of dentistry.

Its pages will be open to any member of the profession who has anything of interest to communicate, and it invites a free expression of views on all subjects, medical or dental.

As each of the gentlemen forming the association is practically a member of the editorial corps, it will have abundant opportunity for collecting all the latest professional news and intelligence.

It will have a body of regular contributors, selected from the very best writers in the profession, and will thus be certain of an abundance of interesting original matter.

It will pay especial attention to reports of the meetings of dental societies, and its aim will be to publish an epitome of all important discussions of professional matters.

It will contain original abstracts and translations of all that is of moment in the foreign journals.

Its profits, if any, will be devoted to its improvement by adding needed illustrations, paying for valuable original communications, and enlarging its size whenever this becomes advisable.

To the accomplishment of the work thus outlined (necessarily relying upon the hearty approval of an appreciative profession), each of the publishers of the INDEPENDENT PRACTITIONER has pledged his best efforts.

Editorial communications, and everything relating to the subscription department, should be addressed to DR. W. C. BARRETT, No. 208 Franklin St., Buffalo, N. Y. Send all other business letters to DR. WILLIAM CARR, 35 West Forty-Sixth Street, New York.

| | | |
|---|---|---|
| ABBOTT, FRANK. . N. Y. | CARR, WILLIAM . . . . . New York. | HILL, O. E. . . . . . Brooklyn. |
| BARRETT, W. C. Buffalo. | DUDLEY, A. M. . . . . . Salem, Mass. | MILLER, W. D. Berlin, Ger. |
| BODECKER, C.F.W. N.Y. | FRANCIS, C. E. . . . . . New York. | PALMER, S. B. . . Syracuse. |

